# Metastasis of the liver with a granulosa cell tumor of the ovary: A case report

**DOI:** 10.3892/ol.2014.2784

**Published:** 2014-12-09

**Authors:** SHUIPING YU, XUELING ZHOU, BINZONG HOU, BO TANG, JIE HU, SONGQING HE

**Affiliations:** 1 Department of General Surgery, The Affiliated Hospital of Guilin Medical University, Guilin, Guangxi 541000, P.R. China; 2 Department of General Surgery, The Fifth Affiliated Hospital of Sun Yat-Sen University, Zhuhai, Guangdong 519000, P.R. China; 3 Department of Pathology, The Affiliated Hospital of Guilin Medical University, Guilin, Guangxi 541000, P.R. China

**Keywords:** metastasis, liver, granulosa cell tumor, ovary

## Abstract

The present study describes the case of a 62 year-old female patient with a metastatic tumor in the right hemi-liver of >25 cm in diameter, who presented to The Affiliated Hospital of Guilin Medical University (Guangxi, China) with acute abdominal pain and severe malnutrition. Radical surgery was performed to remove the tumor by open surgery. A biopsy was not performed prior to the surgery, so the tumor was diagnosed as end-stage primary liver cancer (PLC) based solely on the character and appearance of the tumor on computed tomography prior to surgery. However, subsequent to the surgery, upon analysis by the Department of Pathology, the mass was identified as an ovarian granulosa cell tumor (GCT). These tumors occur rarely, representing only 2–3% of all ovarian tumors, and are well known for late recurrences, with an incidence of 25–30%. As metastasis of the liver with GCT is extremely rare and the data available on the subject is limited by the small number of studies, and due to the absence of a biopsy report prior to surgery, the patient was initially misdiagnosed with PLC. However, despite this misdiagnosis, a good result was obtained, as the patient was later diagnosed with GCT following a detailed pathological examination and was treated with rational therapy. The performance status and quality of life were significantly improved, and the patient remains disease-free at one year post-surgery.

## Introduction

Granulosa cell tumors (GCTs) are rare sex cord-stromal tumors, and are classified into either adult or juvenile forms and the median age at presentation, for the adult form is 50 years. GCTs are low-grade neoplasms, whose early symptoms are uterine bleeding and pain, in addition to pressure symptoms with a palpable mass (1). GCTs have a low malignant potential and a strong tendency for late recurrences, with an incidence of 25–30%. However, hepatic metastases are rare and account for only 5–6% of all GCT recurrences (2,3). These rare metastases usually occupy a wide region of the liver parenchyma as a result of their large size and may be identified by microscopy due to the presence of Call-Exner bodies (4). The first case was reported in the English literature Margolin et al in 1985 (5). As few studies and little data are available on the subject of metastasis in the liver with a GCT of the ovary, a metastasis occurring from GCT of the ovary can easily be misdiagnosed as end-stage PLC, for which surgery may not necessarily be performed, leading to a deteriorative pathogenetic condition. Resectioning liver metastases for GCTs is usually performed only as a palliative procedure rather than as a therapeutic plan, however it may significantly improve the quality of life for the patient (6). The present study reports the case of a patient in whom surgery for GCT of the ovary was performed >20 years prior to recurrence, following which, a second surgery was performed that resulted in a significantly improved quality of life. The patient provided written informed consent.

## Case report

**Figure 1:**
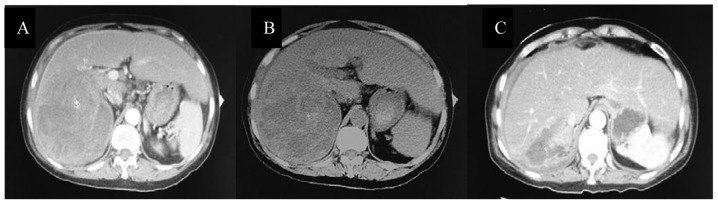
(A) Contrast-enhanced CT of the abdomen showing an enhancing mass (arrow) in the right half of the liver prior to surgery. (B) Delayed-phase CT of the abdomen showing a mass (arrow) of homogeneous lower attenuation in the right half of the liver prior to surgery. (C) Contrast-enhanced CT of the abdomen showing that the mass was no longer present in the right hemi-liver post-operatively. CT, computed tomography.

**Figure 2:**
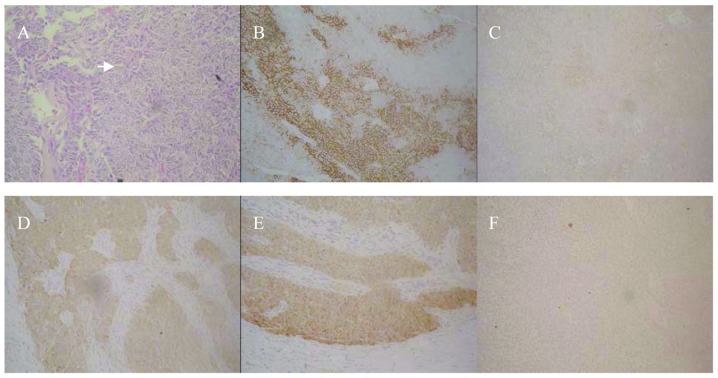
(A) Microscopic findings showing the structure of Call-Exner bodies (arrow) in the tumor tissue of the right hemi-liver. Immunohistochemistry in the tumor tissue of the right hemi-liver was (B) cluster of differentiation (CD)56(+), (C) CD99(+), (D) α-inhibin(+), (E) S-100(+) and (F) cytokeratin19(−) by EnVision staining procedures.

A 62-year-old female was admitted to The Affiliated Hospital of Guilin Medical University (Guilin, China) in 2013 with acute abdominal pain and severe malnutrition. Previously, in 1986, at 35 years of age, the patient had undergone a total abdominal hysterectomy and bilateral salpingo-oophorectomy (TAH+BSO) for a stage 1 grade 1 adult GCT of the ovary in the Second Hospital of Guangxi Province (Guilin, China). The patient did not receive any adjuvant chemotherapy and remained disease-free until 2013. Upon admittance to hospital in 2013, the blood test for the α-fetoprotein (AFP) tumor marker was negative. A computed tomography (CT) scan of the patient was performed and reviewed. A 2.5-mm slightly enhancing mass was observed in the tumor of metastasis; the tumor was ~10×15×25 cm in size (Fig. 1). A biopsy was not obtained prior to surgery. Following the diagnosis of right PLC, surgery was performed. Recurrences were present on the right hemi-liver and jejunum, with sparse nodules. A radical hepatectomy involving segments 5/6, a cholecystectomy and a segmental jejunectomy were performed. Following the resection, the tissues were delivered to the Department of Pathology, and then embedded in paraffin and sectioned. The pathological results showed Call-Exner bodies as microfollicular structures and clear metastasis of the liver, with GCT of the ovary (Fig. 2). Immunhistochemistry results revealed positivite staining for CD56, CD99, inhibin-α and S-100 and negative staining for CK19. The patient made a good recovery, with resolution of the previous abdominal pain, and remains disease-free at one year post-surgery.

## Discussion

Ovarian cancer has the fifth highest mortality rate of all cancers in females, after breast, bowel, lung and uterine cancer, representing 5–6% of cancer-related mortalities (2). In total, 85% of ovarian cancers arise from the ovarian surface epithelium; sex cord-stromal tumors account for 2–5% overall, with GCT being the most common (3). The main characteristics of GCT are the presence of Call-Exner bodies as microfollicular structures on microscopy, and immunohistochemistry results showing positive CD56, CD99, inhibin-α and S-100 staining, but negative CK19 staining. GCTs are generally low-grade neoplasms associated with a long disease-free interval due to the indolent nature of the disease, however, the majority of patients must be manage their condition and be aware of new symptoms, as the tumors are well known late recurrences, which occur with an incidence of 25–30% (4). Hepatic metastases rarely occur, with an incidence of 5–6% of all GCT recurrences (7). The occurrence of these metastases in only one segment is also rare, as they are almost always large in size and occupy a wide region of the liver parenchyma (8).

It may be difficult to differentiate GCT from PLC prior to surgery. In the present study, the patient underwent a TAH+BSO for stage 1 grade 1 GCT in 1986, and no adjuvant chemotherapy was administered. The patient remained disease-free for >20 years until recurrence, which presented as abdominal pain and a large mass in the liver. Consequently, it is important that patients with GCT should be followed up regularly, even if the disease-free interval is long, and that adjuvant treatments may be reserved for patients with large residual or inoperable tumors. The literature on GCTs commonly advocates the use of radiofrequency ablation for hepatic metastases from GCT (9–11). Historically, surgical resection of liver metastases for GCT was performed merely as a palliative procedure, and not as a planned intervention, even though it resulted in a significant increase in disease-free survival (6). We believe that the surgical resection of hepatic metastases for GCT is necessary, particularly in patients who experience a long period of disease-free survival following the primary surgery. Although the surgery has certain risk factors, patients may make a good recovery, with resolution of any previous discomfort, and resulting in another long disease-free period post-operatively.

In the present study, the patient initially presented in 2013 with acute abdominal pain and severe malnutrition. A biopsy was not performed prior to surgery. A metastatic tumor with a maximum diameter of >25 cm was detected, and even though the mass was misdiagnosed as a PLC, surgery was performed to remove the tumor. The patient made a good recovery and remains disease-free at present.

Although the present reported case is rare, it indicates the role of surgical resection for hepatic metastases of GCT, particularly in patients with a record of a long disease-free period. Doctors, and specifically hepatobiliary surgeons, should be aware that patients with GCT should be regularly followed up, even if the disease-free interval is long. Hepatic resection for GCT may significantly improve a patient’s survival time and quality of life.
